# The Value in Science-Art Partnerships for Science Education and Science Communication

**DOI:** 10.1523/ENEURO.0238-20.2020

**Published:** 2020-07-15

**Authors:** Cristian Zaelzer

**Affiliations:** 1 Centre for Research in Neuroscience, Research Institute of the McGill University Health Centre, Montreal H3G 1A4, Quebec, Canada; 2 Department of Design and Computation Arts, Faculty of Fine Arts, Concordia University, Montreal H3G 1M8, Quebec, Canada; 3 The Convergence Initiative, Longueuil J4G 2S1, Quebec, Canada

**Keywords:** collaborations, emotionality, fine arts, graphic design, science communication, science education

## Abstract

Just a fraction of the scientific knowledge produced in laboratories reaches a lay audience. Most of our communication with the public gets lost in translation because of the difficulties that science communication poses to scientists. Among other obstacles, differential exposure to scientific and critical thinking, discrepancies with social narratives, and communication training based in the deficit model add on top of a practice established on avoiding emotionality. In this context, effective communication requires the use of emotions, which are crucial to establishing trust. This commentary provides a rationale for collaboration with graphic design and fine arts to use emotions in science communication and education. It starts by proposing the two-way engagement model as a replacement for the deficit model. Next, it offers a neuroscientific basis for the use of emotions in establishing trust. Finally, it finishes profiling the Convergence Initiative’s efforts to establish bridges across disciplines and communicating science with the public through art.

## Abstract (precis)

Just a fraction of the scientific knowledge produced in laboratories reaches a lay audience. Most of our communication with the public gets lost in translation because of the difficulties that science communication poses to scientists. Among other obstacles, differential exposure to scientific and critical thinking, discrepancies with social narratives, and communication training based in the deficit model add on top of a practice established on avoiding emotionality.

## Significance Statement

Collaborations between neuroscience, graphic design, and fine arts can help scientists to make their findings accessible to the general public through emotionality.

## The Problem of Contemporary Science Communication

With headlines that present ongoing research as conclusive, it is not surprising that most of our communication to the public gets lost in translation, leading readers to forget that science is a dynamic knowledge-building process. Indeed, just a fraction of the scientific knowledge produced in laboratories reaches a lay audience. The many contributing factors for this gap orbit around the difficulty that science communication poses to scientists. Scientific training can take between two to 12 years to complete. In that time, different exposure to scientific knowledge, critical thinking, and social narratives creates huge disparities between trainees and the general public. There are often discrepancies between what scientists think is important, what scientists think the public perceives as important, and what is actually important to the public (Besley and Nisbet, 2013; Besley et al., 2015; Llorente et al., 2019). The lack of training in science communication and lack of incentive to engage in science outreach adds to the gap. We may salute those performing scicomm (short for science communication), but practical actions compensating their efforts, like salaries or tenure merits, do not reflect the importance of their work as a key component in contemporary scientific outreach (Wilsdon et al., 2005; Ngumbi, 2018). Furthermore, science communicators often approach training based on the deficit model, the notion that the public has a knowledge gap, and that scientists only need to fill this gap through education (National Academies of Sciences, Engineering, and Medicine, 2017). They also deal with some general cognitive biases, among those, the knowledge paradox, which states that the more you know, the less you can clearly explain it, and the general confirmation bias, the tendency to favor information that affirms one’s prior beliefs or hypotheses (Lord et al., 1979; Gorman and Gorman, 2017; Goldberg and Hanlon, 2019). These three caveats make the reaching of non-scientific audiences a task far from simple.

Adding to these training issues, the rigorous objective nature of the scientific method demands the avoidance of the emotion that is foundational to human nature. Since Mary Shelley’s *Frankenstein*, the arts have criticized the scientific method and warned of the moral implications of an emotionless practice of science (Ruttkay, 2020). While there has recently been some recognition of the benefits for scientists to acknowledge their attitudes toward science, avoiding emotions has placed us in an uncomfortable position where effective communication requires the use of emotions (Birney, 2013; Gorman and Gorman, 2017; Nature’s Editorial, 2017). In this commentary, I provide rationale for collaboration with graphic design and fine arts to use emotions in science communication and education. I start by proposing a horizontal and reciprocal model of sharing and learning information between participants, the two-way engagement model, as a replacement for the deficit model. Next, I offer a neuroscientific basis for the use of emotions in establishing trust. I finish the commentary by profiling the Convergence Initiative’s efforts to establish collaboration between neuroscience, graphic design, and the arts to create bridges across disciplines and to communicate science with the public through art.

## Education and Neuroscience Views on Engagement

Most scientists disseminate their findings only to peers. About half of them do not perform public outreach at all, considering it to be ineffective. The other half does minimal outreach, mainly focusing on school children, and implicitly adopting the deficit model (Aikenhead, 2006; Besley et al., 2015; Rainie et al., 2015; National Academies of Sciences, Engineering, and Medicine, 2017). Researchers in the field of science communication have repeatedly found the deficit model to be futile, contending that the model is useless in dealing with the “confirmation bias” (Lord et al., 1979; Gorman and Gorman, 2017; Mercier and Sperber, 2017; Sloman and Fernback, 2017). Effective evidence-based decisions require continuous reassessment of information by policymakers. Dynamic engagement between knowledge-producers and individuals, communities, and societies is necessary for successful knowledge translation. Therefore, the deficit model is outdated, and scientists must embrace a practice that gives the public a voice throughout the scientific process. Such a practice can be found in the two-way engagement model (Cooper, 2016). In their 2014 report on scientific culture, the Council of Canadian Academies concluded that the participatory nature of two-way engagement can foster public interest and community engagement “strengthening policy outcomes by pulling in more voices, building support for science, growing interest among youth, encouraging science careers, improving science knowledge, and boosting the overall value of science to society.” (Council of Canadian Academies, Expert Panel on the State of Canada’s Science Culture, 2014). The horizontal didactic techniques practiced in two-way engagement not only help the public to better understand specific scientific issues, but also encourages the audience to realize their active role in advancing scientific knowledge (Bonney et al., 2009; Hallmann et al., 2017; Vogel, 2017; Seibold et al., 2019). Further, two-way engagement ensures a more comprehensive practice of diversity and inclusion by allowing unprivileged and minority voices to be added to the opinions of those constructing and practicing science (Steering Committee for a National Science Communication Strategy (Australia), 2010; Singer et al., 2015; Wilsdon et al., 2005). Public two-way engagement can be achieved in many different ways, but the level of effectiveness increases when the activity is developed over time (Crooks et al., 2017). Longer engagement periods create more opportunities for the public to participate in the process. The participation gives the public a chance to challenge and be critical of the science generating more interest and understanding of the specific question, the scientific inquiry, and the collaborative process. The longer engagement also helps the scientists to understand better the needs from those involved by spending more time and interest with those whom the science could benefit (Powell and Colin, 2008; Nature’s Editorial, 2017). Two-way engagement reaches the public at a personal level, building trust and reciprocity that is essential when communicating a scientific message (National Academies of Sciences, Engineering, and Medicine, 2017).

The use of different neuroscience methodologies has revealed that the interactions of the limbic system circuits with the prefrontal cortex (PFC) and the orbitofrontal cortex (OFC) are critical for our balanced experience of reason and emotions. The balance between the excitation and inhibition of these areas is crucial for decision-making in social settings (DeRubeis et al., 2008; Rilling and Sanfey, 2011; Aggleton et al., 2015). I believe that these brain areas are also involved in how we communicate science and how the public perceives this communication. For example, earlier I discussed trust as a critical contributor to reciprocity in social interactions, being especially important in the efficacy of communication. Trust is not an exclusively rational activity; emotional states have the power to influence and modulate trust judgements based on facial expressions (Schoorman et al., 2007; Caulfield et al., 2016), and the recognition of those emotions is impaired following bilateral damage to the human amygdala (Adolphs et al., 1994). The amygdala is part of the limbic system and is associated with memory and decision-making under social pressure (Edelson et al., 2011, 2014). Although the function of the amygdala has classically been linked to fear, it has recently been shown to be active in the perception of aesthetic experiences (Ikeda et al., 2015; Jacobs and Cornelissen, 2017). Interestingly, cumulative evidence supports a modulatory role of oxytocin (OT) on the amygdala suggesting that trust may involve OT-mediated suppression of the amygdala, thereby reducing the accompanying fear that amygdalar activation produces (Rilling and Sanfey, 2011; Radke et al., 2017; Lopatina et al., 2018). Studies addressing the role of OT on social behavior have shown that people who received a nasal dose of OT spray were more trusting of a social partner that those who did not (Kosfeld et al., 2005). This effect was contextually-dependent on the trustworthiness of the social partner (Mikolajczak et al., 2010). In the rat medial PFC (mPFC), OT attenuates anxiety-like behavior by direct action on the OT receptor (OXTR) as shown by the use of an antagonist to the OXTR, and the lack of OXTR gene (Nakajima et al., 2014; Sabihi et al., 2014, 2017). In humans, genetic polymorphisms for the OXTR are associated with human prosocial decision making (Israel et al., 2009).

The insula, another area of the limbic system, has been linked to aversive behavior, and is known to modulate the activity of the ventromedial PFC (VMPFC) in a social context of decision making (Rilling and Sanfey, 2011). The insula is also active during aesthetic experience, being linked to the valence given to an art piece (Chatterjee and Vartanian, 2016). Other studies have established reciprocity between regions that process reward, emotional appraisal, value, and social behavior (Rilling and Sanfey, 2011). Interestingly, the study of the neural substrates of aesthetics experience by the field of neuroaesthetics has shown activation of the reward circuits and the default mode network (DMN) while individuals experience art. The DMN is a large scale brain network that displays high correlated activity in certain circumstances like mind wandering, day dreaming, external task performance, empathic and metacognitive thinking, projection, and memory recall (Vessel et al., 2012; Chatterjee and Vartanian, 2016; Belfi et al., 2019). In response to music, for example, the dorsal and ventral striata respond to moments of peak pleasure with dopamine release (Blood and Zatorre, 2001; Salimpoor et al., 2011).

Such experiments may be able to explain why different authors state that appealing to emotions can be more influential when explaining and discussing scientific facts (Kolbert, 2017; Requarth, 2017; Saffran et al., 2020). It is easy to imagine a scenario where an aesthetic experience could act in the amygdala, insula, or dopaminergic reward system to then modulate “rational” and decision-making areas in the PFC and OFC, thereby priming individuals to receive a message.

## The Use of Emotionality to Communicate Information to Humans. The Path from Design to Arts

Because of the direct relationship between art and emotion, the recruitment of art and design in science communication assists the public to situate themselves in the complexities of scientific inquiry. Similarly, art in popular culture has a strong influence in shaping people’s perception of science and scientists. Films, novels, comics, and illustrations are usually more appealing, eye catching, and memorable than formal scientific lectures (Van Riper, 2003).

Over the last 30 years, several prominent organizations have demonstrated the effectiveness of art in two-way engagement. The United Kingdom Wellcome Trust’s Sciart program (1996–2006), with 118 projects and nearly £3 million in funding, illustrated how art can help to promote scientific knowledge in new and more attractive ways (Glinkowski and Bamford, 2009). ArtNeuro, at the University of London, showcased hard-to-understand science in a free public art exhibitions format making science more accessible to the public in a way not explored before (Bryony, 2014). In the United States, initiatives like the MIT Media Labs (1985), The Story Collider (2010), Northwest NOGGIN (2012), and Reclamation (2016) use storytelling, art education, and media design to involve the public in the scientific process. Many groups in the United States advocate for STEM programs to become STEAM programs by incorporating the arts in an effort to “achieve a synergistic balance.” Studies examining the outcomes of art-science collaborations have described increased observational and analytic prowess, better questioning skills, more focused periods of intense concentration, and greater understanding that problems can have multiple answers (Piro, 2010).

**Figure 1.**
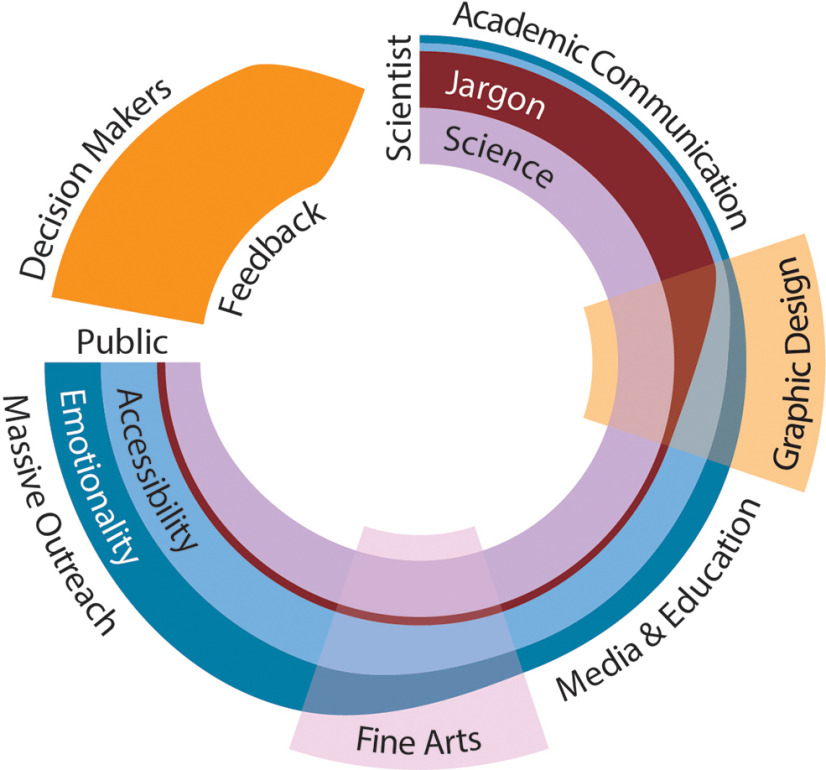
Accessibility and emotionality in science education and communication. The figure illustrates the process of accessibility to science, starting with scientists and his findings finishing with the general public and their feedback to the scientists. From a communicative perspective, scientific findings can be studied under four components divided into two groups. Group one: science, comprising all the aspects of the method, and jargon, defined as the set of highly specialized codes to communicate science to peers. Group two: accessibility, defined as the degree of access for non-scientist to scientific findings, and emotionality, defined as the emotional cues involved in proper communication. The model proposes collaborative efforts where graphic design and fine arts act as filters changing the value of one or more of these parameters. Previous to graphic design, the communication of science is done only among peers in scientific conferences and close door collaborations, jargon here is essential and relevant. Graphic design reduces the jargon translating knowledge to visual forms adding emotionality to the result. The product can be used for media and education before reaching the next filter, fine arts. Fine arts maximize the emotional aspects of communication, increasing accessibility for the public. The information now is ready for massive outreach and discussion. Once it arrives at the public, the process has reduced some of the unnecessary scientific aspects by removing jargon almost entirely and the irrelevant details from the science. That said, science still is at the core of the diagram keeping a relevant proportion of key aspects balancing scientific and public interest. These aspects can include, among others, results, methods, processes, and practitioners. Since the model is collaborative, the start and end differ in thickness, being the side of the public thicker illustrating the added value of design and fine art collaborations. The last aspect of the model is the feedback to scientists, which includes knowledge-stakeholders and decision makers. Scientists should consider this step as crucial to adding the value of science to society. We think that the perpetuation of scientific work as independent enterprise damage that step and hurt the practice of science.

In practice, I think connecting emotions to scientific discoveries involves at least two steps (Fig. 1). First, requires a translational mechanism that sheds away the scientific jargon to leave a simple, not a simplistic, accessible visual form. Graphic design can advance scientific communication one step further to an emotive provocation. Like the Rosetta Stone of science communication, graphic design is able to translate complex scientific concepts into visual language. Interestingly, graphic design and the scientific method share in their praxis, the design process iteratively refines communication problems in a manner similar to how the scientific method progresses through hypothesis, experimentation, and analysis. In design, the outcome materializes as a visual solution. The use of elements such as typography, iconography, photography, and illustration, arranged and organized in layouts and visual hierarchy, exemplify the well-known Gestalt principle, “the whole is more than the sum of its parts.”

Second, using the visual form produced in step one as the new starting point, the communicators invite fine art practitioners to a two-way engagement conversation with scientists. Collaborating with artists gives scientists the chance to draw on innovative techniques to impart scientific concepts with an emotional appeal. From the neuroscience perspective, before any possible conscious reasoning takes place, the limbic system judges experiences as aversive, desirable, or rewarding. Emotions act as cues to summon memories from past events, influencing our behavior to avoid or approach new situations, or in this case, new information. Fine arts practitioners are instrumental in the process of modeling the emotive message to give science the right tone at the moment of communication. The variety and richness of approaches used by artists can help scientists connect to audiences’ narratives and transform our teaching methods into inclusive and attractive environments while paying homage to the human aspect of the scientific endeavor. We learn more about things we like, things that surprise us, things that connect to us, things that stand apart from the daily noise of everyday life.

## Exploring New Methodologies to Communicate Science, Partnership with the Arts and Design. The Convergence Initiative

In 2016, I founded The Convergence Initiative as an organization committed to bridging neuroscience, design, and the arts. A primary activity of The Convergence Initiative has been the development and organization of a two-semester course that integrates topics in the disciplines of design, fine arts, and neuroscience. Co-designed with art educator, sci-art practitioner, and researcher Bettina Forget, the course facilitates a year-long collaboration between neuroscience graduate students from McGill University and art and design students from Concordia University’s Faculty of Fine Arts. Instead of enlisting scientists as artistic muses (like some SciArt collaborations), we encourage a horizontal partnership between the art and science students from day one to the completion of the eight-month collaboration.

Using a two-way engagement approach as much as possible, we start the program by mixing our students in the same class. Neuroscience graduate students and undergraduates in fine arts and design prepare introductory exposition using the Ignite format. This presentation consists of 20 slides, which automatically advance every 15 s, creating a fast and fun 5-min presentation. These presentations start a selection process where the students form pairs by the end of the first semester. In the second semester, these pairs collaborate to develop innovative sci-art projects. During the first semester, we deliver basic concepts in neuroscience using interactive presentations, games, and experiments. We pair an artistic activity with each topic. For example, our class on the visual system is followed by a class in the theory of color where students explore the ideas of Josef Albers on the interaction of pigments. Outside of the classroom, the course includes site visits, where artists and scientists have the chance explore each other’s exclusive silos (the laboratories and technological facilities of the sciences and the studios, museums, and galleries of the art-world). Discussion is a requirement, and a substantial part of the class involves comparing the practices of science and art. The revision of the philosophical bases of the scientific practice and the debate of those ideas in private and public forums is also part of our program.

Theoretical and practical aspects of science communication are covered side by side with non-traditional methods for science delivery. Participants explore topics such as the neuroscience of interactions between reason and emotion alongside graphic design, zine publication, podcast production, and storytelling. Cognitive bias, complexity avoidance, risk perception, as well as group psychology, are also discussed. The culmination of this training is a public science symposium where the neuroscience students use these tools and others to present their research topic in an innovative way. Over the years, our students have found numerous creative ways to communicate their research science. To mention just a few, these tools have included comics, musical composition, storytelling, and sculpture.

The course concludes with a month and a half long public exhibition of art inspired by neuroscience. The art pieces are developed on the research done by each team of artists and neuroscientists during a complete semester. This process starts with the scientific research contributed by the neurosciences, followed by the artists guided inquire about aspects of the scientific process that are less obvious to the scientists. To mention a few, the teams examine the impact and purpose of the research, relation between human and non-human aspects, probable futures and questions raised by the findings, and the ethical aspects versus the benefits for the global community. The teams then established the research questions and chose the medium, materiality, and methodology to create art that explores that query. In the process, the teams ensure that the scientific aspects are respected while allowing the fluidity of the creative proceedings. Both scientists and artists are strongly encouraged to participate and explore their counterparts’ fields. This active sharing and exploration are reflected on the shared authorship of the art pieces, with no obvious distinctions on their backgrounds. By this point, we have guided our students from the first scientist-artist encounter using the two-way engagement program to produce a final multidisciplinary science-art collaboration reflected in the art pieces now delivered to the visitors of the exhibitions.

Our program owes part of its success to the constant support of institutions like the Faculty of Fine Arts at Concordia University (who hosts the course), the Research Institute of the McGill University Health Centre (RI-MUHC) and its Brain Repair and Integrative Neuroscience Program (BRaIN), and the Integrated Program in Neuroscience of McGill University. This interdisciplinary support highlights the crucial role that other stakeholders play in the program, by contributing facilities, personnel, expertise, and funding.

Some of the most important outcomes of the course are the new perspectives that the neuroscience students take with them. Insight into how other people in society think and operate. New understanding on the attitude of outsiders toward science. What “think outside of the box” really means. A deep appreciation for synergetic collaboration. A return to the playground.

In order for scientists to connect with others, they must be flexible between feeling and reason. We must maintain an unbiased approach toward the observation of nature, but when communicating with the public, we must maintain our humanity. The benefits are quite obvious– improved appreciation for the scientific endeavor, increased public trust in science, increased scientific funding, political support for representatives who use evidence to inform their public policies. The problems that humanity faces today are multifaceted; as such, they require the expertise and contribution of many partners to reach the solutions. Science is just one aspect of that solution, recognize the help and contribution of others can help us to create better spaces to develop those future multifaceted thinkers that will finally bring us one step closer to a better world. Collaborations between scientists, designers, and artists are then more important than ever in the construction of that new fabric.

## Synthesis

Reviewing Editor: Lynda Erskine, University of Aberdeen Decisions are customarily a result of the Reviewing Editor and the peer reviewers coming together and discussing their recommendations until a consensus is reached. When revisions are invited, a fact-based synthesis statement explaining their decision and outlining what is needed to prepare a revision will be listed below. The following reviewer(s) agreed to reveal their identity: NONE.

This is an interesting and thought-provoking article. It is likely to be of wide general interest. However, there are a few places in the manuscript where the terminology/meaning of phrases could be clarified to ensure the manuscript is understandable by the widest possible audience.

1. Lines 37 - 39. “Furthermore, science communicators often approach training based on the deficit model and cognitive biases like the knowledge paradox and the confirmation bias difficulting the reaching of non-scientific audiences"

To aid understanding, it would be good here to clarify the specific meaning of some of the terms and phrases used in this sentence, in particular:

- “deficit model” (Would be good to explain what exactly meant by this here rather than later in the manuscript).

- “knowledge paradox” What exactly is meant by this phrase? - it was not clear to me"

- “confirmation bias” - what exactly is meant by this term? (Having read down I see this is explained later - be helpful to move this explanation here where term first used).

2. Lines 90 - 92: “The use of different neuroscience methodologies has revealed the interactions between both the prefrontal (PFC) and the orbitofrontal (OFC) cortices with the limbic system circuits, to be the key for our experience of reason and emotions respectively”.

I was not 100% certain if the last part of the sentence (“key for our experience of reason and emotions respectively” refers to the prefrontal and orbitofrontal cortices or prefrontal/orbitofrontal cortices versus limbic circuits. It would be helpful to clarify this.

3. Line 94 I did not really understand what was meant by “I trust that these brain areas are also...” The use of the word “trust” here seemed a bit out of context. Would “believe” be an appropriate alternative?

4. Line 120 “the default mode network” - it would be helpful to clarify exactly what is meant by this.

5. Line 194 “We pair an artistic activity which each topic” Should “which” be “with”?

## Author Response

Dear Lynda, bellow you will find in blue my detailed responses to your comments. Thank you so much for reviewing it.

---------------------------------------------

Manuscript Instructions

---------------------------------------------

Synthesis of Reviews:

Significance Statement Comments for Author (Required):

N/A

Comments on the Visual Abstract for Author (Required):

N/A

Synthesis Statement for Author (Required):

This is an interesting and thought-provoking article. It is likely to be of wide general interest. However, there are a few places in the manuscript where the terminology/meaning of phrases could be clarified to ensure the manuscript is understandable by the widest possible audience.

Thank you so much for the amazing comments. I am sure the topic is going to spark conversation as it has done hundreds of times in my work putting these principles in practice.

1. Lines 37 - 39. “Furthermore, science communicators often approach training based on the deficit model and cognitive biases like the knowledge paradox and the confirmation bias difficulting the reaching of non-scientific audiences"

To aid understanding, it would be good here to clarify the specific meaning of some of the terms and phrases used in this sentence, in particular:

- “deficit model” (Would be good to explain what exactly meant by this here rather than later in the manuscript).

- “knowledge paradox” What exactly is meant by this phrase? - it was not clear to me"

- “confirmation bias” - what exactly is meant by this term? (Having read down I see this is explained later - be helpful to move this explanation here where term first used).

I agree with all the suggestions, I have move some of the definitions to this area to clarify and help the reader instead of holding it confused while advance in the text. I also add a definition for the knowledge paradox. Here is how the text looks now:

Furthermore, science communicators often approach training based on the deficit model, the notion that the public has a knowledge gap, and that scientists only need to fill this gap through education (National Academies of Sciences, 2016). They also deal with some general cognitive biases, among those, the knowledge paradox, which states that the more you know, the less you can clearly explain it, and the general confirmation bias, the tendency to favour information that affirms one’s prior beliefs or hypotheses (Goldberg and Hanlon, 2019; Gorman and Gorman, 2017; Lord et al., 1981). These three caveats make the reaching of non-scientific audiences a task far from simple.

2. Lines 90 - 92: “The use of different neuroscience methodologies has revealed the interactions between both the prefrontal (PFC) and the orbitofrontal (OFC) cortices with the limbic system circuits, to be the key for our experience of reason and emotions respectively”.

I was not 100% certain if the last part of the sentence (“key for our experience of reason and emotions respectively” refers to the prefrontal and orbitofrontal cortices or prefrontal/orbitofrontal cortices versus limbic circuits. It would be helpful to clarify this.

To make this paragraph clearer I have changed to the following:

The use of different neuroscience methodologies has revealed that the interactions of the limbic system circuits with the prefrontal cortex (PFC) and the orbitofrontal cortex (OFC), are critical for our balanced experience of reason and emotions. The balance between the excitation and inhibition of these areas is crucial for decision-making in social settings.

3. Line 94 I did not really understand what was meant by “I trust that these brain areas are also...” The use of the word “trust” here seemed a bit out of context. Would “believe” be an appropriate alternative?

Agreed, I have changed to “believe.”

4. Line 120 “the default mode network” - it would be helpful to clarify exactly what is meant by this.

To add clarity, I have included a brief explanation. The rearranded paragraph now looks like this:

Interestingly, the study of the neural substrates of aesthetics experience by the field of neuroaesthetics has shown activation of the reward circuits and the default mode network (DMN) while individuals experience art. The DMN is a large scale brain network that displays high correlated activity in certain circumstances like mind-wandering, day-dreaming, external task performance, empathic and metacognitive thinking, projection, and memory recall (Belfi et al., 2019; Chatterjee and Vartanian, 2016; Vessel et al., 2012).

5. Line 194 “We pair an artistic activity which each topic” Should “which” be “with”?

Agreed, I have changed to “with.”
